# The role of senescence-related hub genes correlating with immune infiltration in type A aortic dissection: Novel insights based on bioinformatic analysis

**DOI:** 10.1371/journal.pone.0326939

**Published:** 2025-06-25

**Authors:** Wei He, Panli Tang

**Affiliations:** 1 Department of Ophthalmology, Affiliated Hospital of Southwest Medical University, Luzhou, China; 2 Department of Cardiovascular Surgery, The General Hospital of Western Theater Command, Chengdu, China; Brigham and Women's Hospital Division of Cardiovascular Medicine, INDIA

## Abstract

**Background:**

Stanford type A aortic dissection (AAD) is a fatal disease that confers extremely high morbidity and mortality. Cellular senescence, characterized by a permanent cell cycle arrest, has been implicated in the onset and progression of cardiovascular disease and immune cell infiltration has been observed in the aortic walls of dissected specimens. However, the precise mechanisms through which senescent cells interact with immune infiltration to contribute to the development and progression of AAD remain unclear.

**Methods:**

Cellular senescence-related genes (SRGs) were identified via the CellAge database. Patient and normal control datasets (GSE52093 and GSE190635) were retrieved from the Gene Expression Omnibus (GEO) database, with GSE190635 serving as the validation set. Differentially expressed genes (DEGs) linked to AAD were determined from the GSE52093 dataset. We intersected SRGs with DEGs to identify differentially expressed senescence-related genes (DESRGs), which were subsequently analyzed for Gene Ontology (GO) enrichment, Kyoto Encyclopedia of Genes and Genomes (KEGG) pathways, and protein-protein interactions (PPI). Hub DESRGs were selected based on their connectivity degree and diagnostic genes were further refined via gene expression level evaluation and receiver operating characteristic (ROC) curve analysis. Additionally, a miRNA-gene network involving hub DESRGs was constructed. Finally, CIBERSORT was employed to analyze the compositional patterns of the 22 types of immune cell fractions in AAD.

**Results:**

A total of 700 DEGs were identified from the GSE52093 dataset, and 279 SRGs were obtained from the CellAge database. 20 DESRGs, comprising 9 senescence suppressor genes and 11 senescence inducible genes, were identified eventually by overlapping DEGs and SRGs. The top 8 hub DESRGs, including CHEK1, CENPA, FOXM1, BRCA1, AURKA, MAD2L1, PTTG1 and EZH2 were identified. Moreover, three diagnostic genes with high Area Under the Curve (AUC > 0.9) were identified: CHEK1, FOXM1, BRCA1. Additionally, immune cell infiltration analysis revealed correlations between hub DESRGs and CD8 T cells, NK cells, and macrophages. Compared with normal tissues, AAD tissues exhibited a significant decrease in CD8 T cells and an increase in NK cells and macrophages.

**Conclusion:**

Cellular SRGs, such as CHEK1, CENPA, FOXM1, BRCA1, AURKA, MAD2L1,PTTG1 and EZH2 might hold significance in AAD, among which CHEK1, FOXM1, BRCA1 could potentially serve as molecular biomarkers for the diagnosis and treatment of AAD. These genes might contribute significantly to the occurrence and advancement of AAD by modulating the inflammatory response or immune regulation.

## Introduction

Aortic dissection (AD) is a life-threatening condition characterized by tears in the aortic wall. It is classified into two main types: Stanford type A (AAD) and Stanford type B, based on the anatomical location of the primary intimal tear and the extent of the dissection involvement [1]. AAD typically begins in the aortic root or ascending aorta, or both, and rapidly progresses through the entire aorta, posing a significant global mortality risk. Conversely, type B dissections usually originate from the descending thoracic aorta just beyond the branching of the subclavian artery and typically do not affect the ascending aorta. When AAD occurs, timely and accurate diagnosis, along with effective treatment are pivotal in reducing mortality rates, minimizing adverse events, and enhancing overall survival. Currently, the primary pharmacotherapy emphasizes blood pressure and heart rate reduction. However, there are no proven medical therapies that can slow or prevent the progression of AAD [2]. Until now, open surgery stands as the most dependable approach for AAD, which requires advanced technical skills and experienced members of the surgical team within a proficient aortic center. Nonetheless, this presents a challenge in areas with limited medical resources. A registry study from China reported a pharmacotherapy rate of 35.6% but a mortality rate of 42.5% for AAD. While the surgical therapy rate was 52.6%, with a mortality rate of 5.3% [3]. Therefore, conducting in-depth research into the molecular and cellular mechanisms underlying AAD is crucial for the development of innovative pharmacologic treatments and efficient management strategies.

AGEs are strongly associated with cardiovascular diseases such as atherosclerosis and thrombosis [4]. The aging-related pathological mechanism contributing to atherosclerosis primarily encompasses inflammation, endothelial dysfunction, oxidative stress and so on [5]. Cell senescence, a state of robust cell-cycle arrest, is an essential aging phenotype and occurs under severe cellular stress. It plays a critical role in age-related organ dysfunction and can be triggered by aging in various cell types, including endothelial cells, which produce numerous vasoactive factors crucial for preserving tissue homeostasis [6,7]. Endothelial senescence escalates with systemic aging, result in cellular dysfunction and ultimately contributing to the development of cardiovascular diseases, including atherosclerosis [8], which can lead to fatal AAD. Although the study of cellular senescence has made great progress in recent decades. Nevertheless, the precise mechanisms through which senescent cells contribute to the progression of AAD and the pathophysiology between endothelial senescence and AAD are still not very clear. In addition, advancements in bioinformatic technology have unveiled certain hub genes and their interactions associated with AAD [9,10] and some studies have highlighted a distinct connection between AAD and immune-inflammatory mechanisms [11]. Nevertheless, until now, no studies have provided a more comprehensive understanding of immune cell infiltration in AAD or the correlation between immune cells and hub genes based on bioinformatic analysis, which would likely uncover new potential therapeutic targets for AAD.

In this study, we initially retrieved the dataset of GSE52093 from the Gene Expression Omnibus (GEO) database (https://www.ncbi.nlm.nih.gov/gds/?term=) and standardized it using R software (version 3.6.1). Subsequently, we identified DEGs distinguishing AAD patients from control subjects within this dataset. SRGs were downloaded from the CellAge (https://genomics.senescence.info/cells/) database and DESRGs were obtained by intersecting the DEGs and SRGs. Then, we conducted analyses on the DESRGs using bioinformatics techniques, such as Gene Ontology (GO) term enrichment and the Kyoto Encyclopedia of Genes and Genomes (KEGG). A protein-protein interaction (PPI) network of the DESRGs was constructed using STRING database (version12.0, https://string-db.org/), and Cytoscape software (version3.9.1) was used to prettify the PPI network and identify hub genes. Moreover, dataset of GSE190635 was downloaded as a validation set, and ROC curves were used to assess the AUC value and predictive capability of the key DESRGs in these two different datasets. Finally, CIBERSORT was employed to assess variations in the infiltration of 22 immune cell types between AAD tissues and control tissues. Subsequently, we delved deeper into association between key DESRGs and markers of infiltrating immune cells.

## Materials and methods

### Data collection

**Fig 1:**
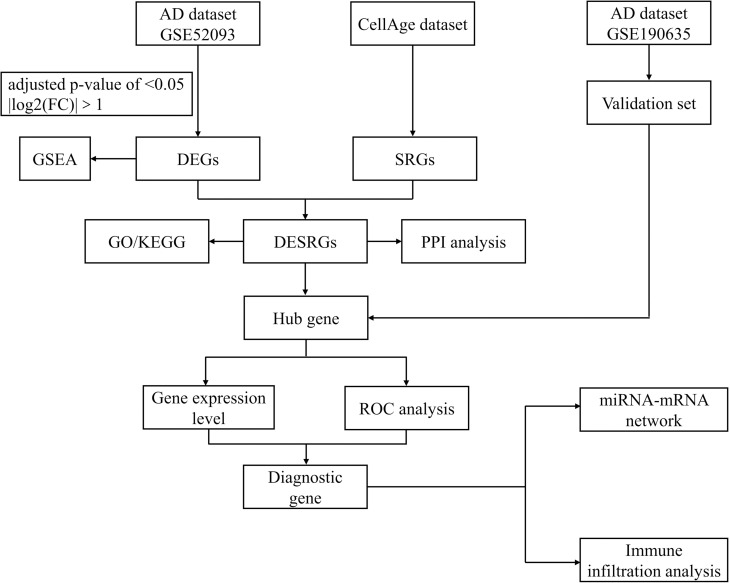
Study flowchart. AD, aortic dissection; FC, fold change; DEGs, differentially expressed genes; SRGs, senescence-related genes; GSEA, gene set enrichment analysis; DESRGs, differentially expressed senescence-related genes; PPI, protein-protein interaction; GO, Gene Ontology; KEGG, Kyoto Encyclopedia of Genes and Genomes; ROC, receiver operating characteristic.

A total of 279 SRGs were download from the CellAge database and the comprehensive list of all SRGs can be found in S1 Table. Then, we conducted a search in the GEO database for datasets containing AAD patients. The inclusion criteria should satisfy the following simultaneously: “aortic dissection”, “homo sapiens”, “expression profiling by array”, and “tissue”. Eventually, we included two datasets in this study: a GPL10558-platform dataset, GSE52093, consisting of 5 normal and 7 AAD samples, and a GPL570-platform dataset, GSE190635, comprising 4 normal and 4 AAD samples. Detailed information for both datasets is outlined in Table 1 and Fig 1 illustrates the flow chart depicting the study’s progression. The study protocol adhered to the principles of the Declaration of Helsinki. This study does not involve human participants or animal research requiring ethical approval. All data analyzed in this study were obtained from publicly available datasets in the GEO database. These datasets are freely available for research purposes and are compliant with ethical and legal standards.

**Table 1:** The fundamental information of the included datasets of AAD from GEO database.

Series	Platform	Contributor	Sample Size(T/N)	Publication	Year	Country
GSE52093	GPL10558	Pan S et al	7/5	–	2014	China
GSE190635	GPL570	Wang X	4/4	–	2021	China

Abbreviations: AAD, type A aortic dissection; GEO, Gene Expression Omnibus

T, Test group; N, Normal group

### Data processing and identification of DEGs and DESRGs between AAD and control

Prior to the analysis of DEGs and DESRGs, the flowing preprocessing steps were performed: first, we computed the average values of probe sites if more than one probe was mapped to the same gene and probes without corresponding gene symbol found were excluded. Second, data were background-corrected and quantile normalized among arrays using the limma package within the R software (version 4.2.0). Last, DESeq2 R package facilitated the analysis of DEGs between patients with AAD and normal controls and genes were considered differentially expressed when exhibiting an adjusted P value < 0.1 and | log_2_FC| > 1 (FC: fold change). Furthermore, Gene Set Enrichment Analysis (GSEA) was conducted utilizing the GSEAPreranked function available in the GSEA software (version 4.1.0) to elucidate the potential mechanism associated with AAD. Of the available gene sets, Hallmarks (h.all.v7.5.1.symbols.gmt), Human phenotype (c5.hpo.v2022.1.Hs.symbols.gmt), Immunologic signatures (c7.all.v2022.1.Hs.symbols.gmt), and All Canonical Pathways (c2.cp.all.v2022.1.Hs.symbols.gmt) were chosen as the reference gene set and package “ggplot2” was used to better visualize the result. Significantly enriched terms were considered with an adjusted P value < 0.05 and a false discovery rate (FDR) < 5%. Finally, DESRGs were obtained by overlapping DEGs and SRGs.

### GO and KEGG pathway enrichment analysis of integrated DESRGs

To investigate the potential biological functions of DESRGs, KEGG pathway analysis and GO enrichment analysis were performed using the “cluster profiler” and “GOplot” packages of R software. The results, which were considered statistically significant if the P value < 0.05, were visually represented through bubble chart and chord plots.

### PPI network construction and verification of hub DESRGs

Utilizing the STRING database, a PPI network was constructed for DESRGs with an interaction score threshold set at > 0.4. Then, the interaction results were downloaded and visualized using Cytoscape software (version 3.9.1). Subsequently, Molecular Complex Detection (MCODE) analysis identified key cluster and Cytohubba plugin was used for screening hub genes by several topological algorithms.

### Validation of diagnostic values of DESRGs

First, ROC curves were constructed utilizing the mRNA expression data from GSE52093, and the AUC values of the hub genes were computed via the pROC package in R software to assess their diagnostic efficacy. Then, hub DESRG expressions in the validation dataset, GSE190635, were extracted and subjected to t-test in R. ROC curve analysis was also conducted, calculating AUCs to determine the predicted capabilities of the hub DESRGs. Hub genes with AUC values greater than 0.9 were considered high-potential diagnostic biomarkers. Statistical significance was defined as P < 0.05.

### Construction of miRNA-gene regulatory network

Analysis of miRNA targeting and networks was conducted using the miRNet database (https://www.mirnet.ca/,version 2.0), and Cytoscape software was used to visualize miRNA-gene interaction network.

### Analysis of immune cell infiltration

Based on gene expression profiles, immune-cell proportions were analyzed by CIBERSORT. First, the LM22 signature gene file encompassing 22 immune cell types, available on the CIBERSORT website (https://cibersortx.stanford.edu/index.php), estimated total immune infiltrate and specific immune cell subsets. Then, a correlation heatmap illustrated Pearson coefficients, delineating relationships among infiltrated immune cells. Scatter plot visually portrayed variations in immune cell infiltration between AAD and control groups. Last, Spearman’s rank-order correlation analysis between hub DESRGs and immune cells was conducted to explore immune mechanisms in AAD development, with results visualized using the “ggplot2” package.

### Statistical analysis

Statistical analyses were performed using R software (version 3.6.1) in our research. Unpaired Student’s t-test or Mann–Whitney U-test was used for comparing gene expression levels between the AAD and normal groups. Gene-gene correlations was performed using Pearson correlation. ROCs curves were utilized to evaluate AUCs and predictive abilities. A significance level of P < 0.05 was set for statistical significance.

## Results

### Screening of DEGs and DESRGs in AAD

**Fig 2:**
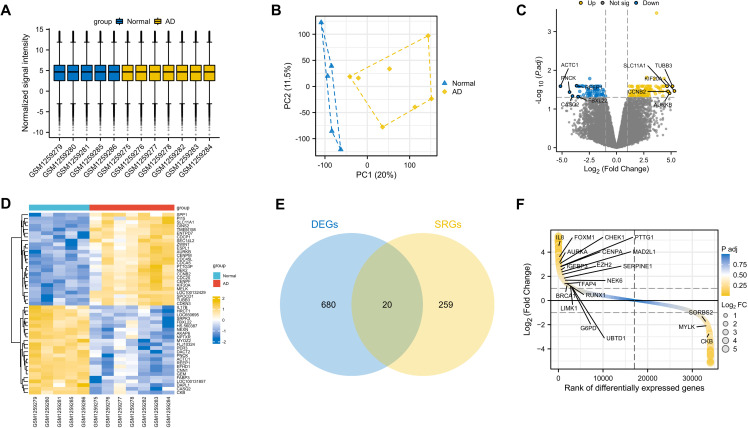
Identification of DEGs and DESRGs of AD in the GSE52093 dataset. (A) Normalized data of GSE52093. (B) PCA plot between AD and normal group. (C) Volcano plot, (D) heatmap, and (E) Venn diagram of overlapping genes between DEGs and CellAge database. (F) Difference ranking plot of the DESRGs. AD, aortic dissection; DEGs, differentially expressed genes; SRGs, senescence-related genes; DESRGs, differentially expressed senescence-related genes; PCA, principal components analysis.

**Fig 3:**
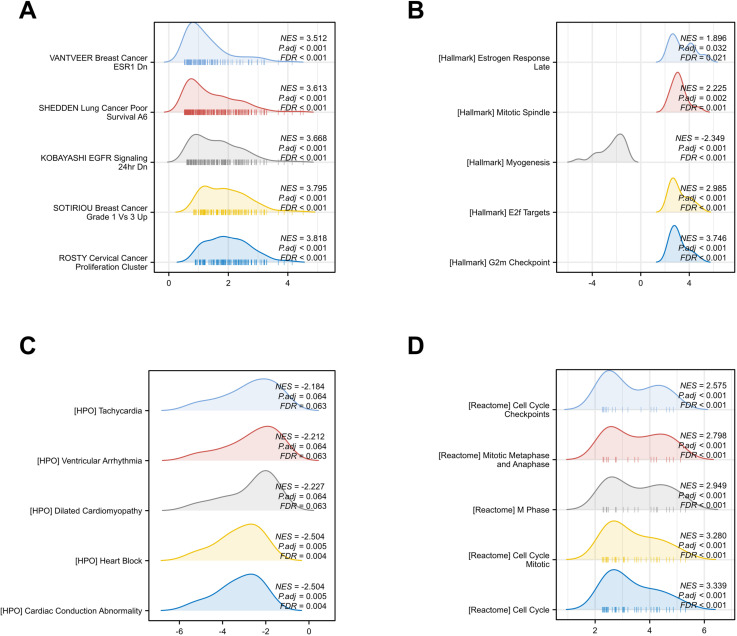
Gene set enrichment analysis (GSEA) of the DEGs. Four gene sets were chosen as the referenced gene set (A) Hallmarks (h.all.v7.5.1.symbols.gmt). (B)Immunologic signatures (c7.all.v2022.1.Hs.symbols.gmt). (C) Human phenotype (c5.hpo.v2022.1.Hs.symbols.gmt). (D) All Canonical Pathways (c2.cp.all.v2022.1.Hs.symbols.gmt). FDR, false discovery rate; DEGs, differentially expressed genes.

**Fig 4:**
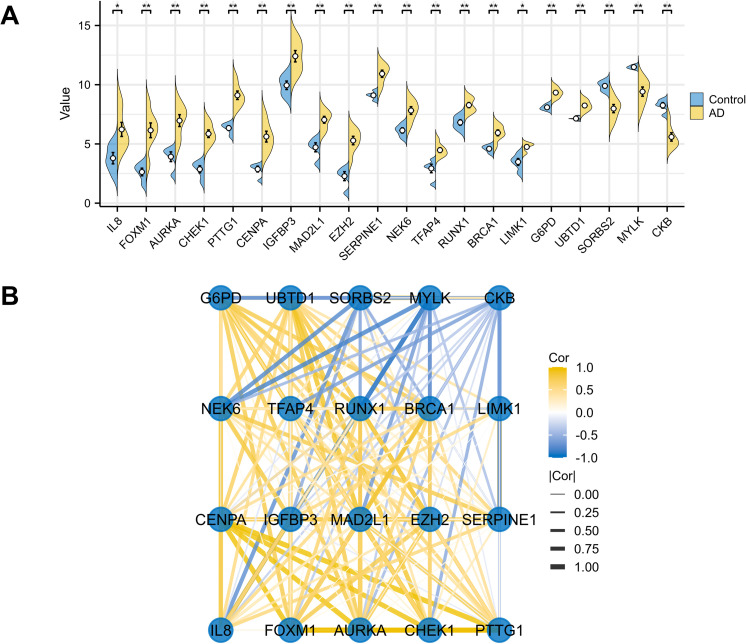
Expression and correlation analysis of 20 DESRGs. (A) Expression levels of DESRGs in the AD and control samples in the GSE52093 dataset were visualized by pods plot. *p < 0.05, **p < 0.01. (B) Spearman correlation among 20 DESRGs. AD, aortic dissection; DESRGs, differentially expressed senescence-related genes.

The expression matrices of the GSE52093 dataset underwent normalization using the R package “limma”. The boxplots exhibited significant linear distribution trends (Fig 2A). The PCA showed obvious differences between the AAD and control group (Fig 2B). A total of 700 DEGs (S2 Table) were identified from GSE52093 with an adjusted P value < 0.1 and | log_2_FC| > 1, consisting of 414 up-regulated and 286 down-regulated genes. The volcano plot exhibited the distinctive distribution of DEGs (Fig 2C) and the heat maps (Fig 2D) illustrated the expression patterns of the 50 up-regulated and down-regulated genes. Ultimately, through the integration of DEGs and SRGs, 20 overlapping genes (DESRGs) were identified for further analysis (Fig 2E), visually highlighted in the differential ranking plot (Fig 2F). Among them, 17 DESRGs showed upregulation, whereas 3 DESRGs demonstrated downregulation. Table 2 contains information regarding the 20 DESRGs and their respective roles in senescence. The GSEA analysis revealed that DEGs mainly participated in the ROSTY Cervical Cancer Proliferation Cluster, Hallmark G2m Checkpoint, HPO Cardiac Conduction Abnormality, and Reactome Cell Cycle among the four reference gene set (Fig 3). The dot plot illustrated the expression levels, while the network plot depicted the correlations among the 20 DESRGs (Fig 4).

**Table 2:** Twenty differentially expressed senescence-related genes.

(17 up-regulated and 3 down-regulated)		
**Gene name**	**Description**	**Senescence effect**
IL8	Interleukin-8	Induces
FOXM1	Forkhead box protein M1	Inhibits
AURKA	Aurora Kinase A	Inhibits
CHEK1	Checkpoint Kinase 1	Induces
PTTG1	Pituitary tumor-transforming gene 1	Induces
CENPA	Centromere protein A	Inhibits
IGFBP3	Insulin like growth factor binding protein 3	Induces
MAD2L1	Mitotic arrest deficient 2 like 1	Inhibits
EZH2	Enhancer of zeste 2 polycomb repressive complex 2 subunit	Inhibits
SERPINE1	Serpin family E member 1	Induces
NEK6	NIMA related kinase 6	Inhibits
TFAP4	Transcription factor AP-4	Induces
RUNX1	Runt-related transcription factor 1	Induces
BRCA1	BRCA1 DNA repair associated	Inhibits
LIMK1	LIM domain kinase 1	Induces
G6PD	Glucose-6-phosphate dehydrogenase	Inhibits
UBTD1	Ubiquitin domain containing 1	Induces
SORBS2	Sorbin and SH3 domain containing 2	Induces
MYLK	Myosin light chain kinase	Inhibits
CKB	Creatine kinase B	Induces

### Functional analysis of integrated DESRGs

**Fig 5:**
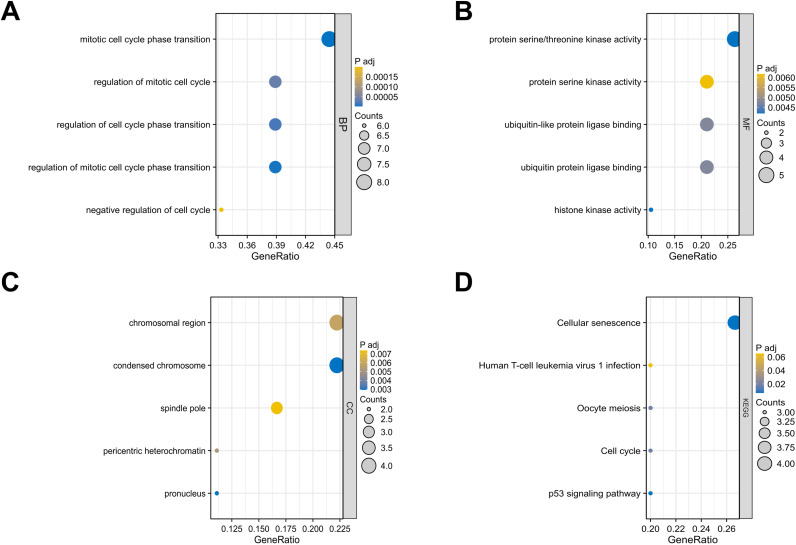
Bubble graphs of 20 DESRG-enriched GO terms and KEGG pathways. (A–D) Represent BP, MF, CC, and KEGG, respectively. GO, Gene Ontology; KEGG, Kyoto Encyclopedia of Genes and Genomes; BP, biological process; CC, cellular component; MF, molecular function; DESRGs, differentially expressed senescence-related genes.

**Fig 6:**
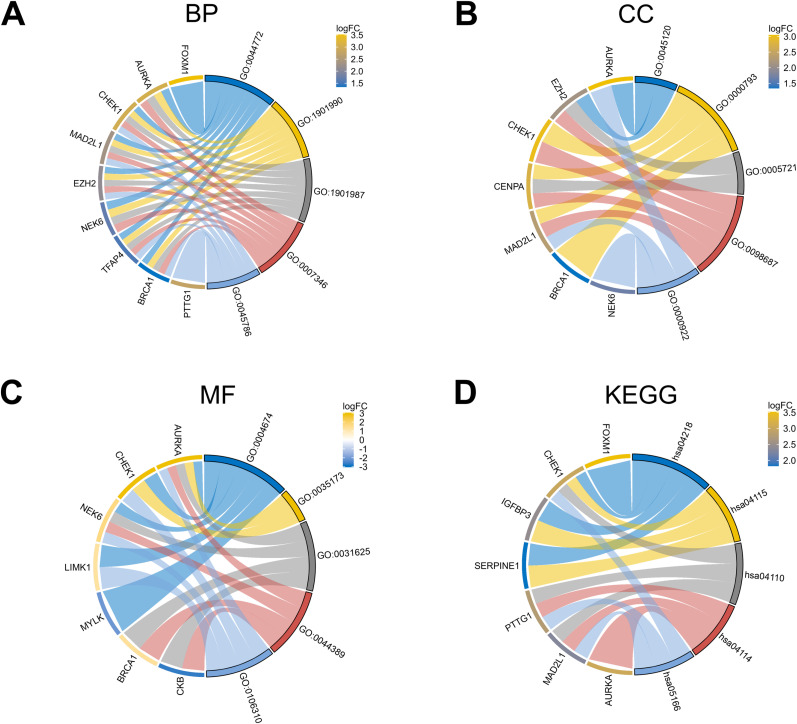
Chord plots of the 20 DESRG-enriched GO terms and KEGG pathways. Panels (A–D) represent the top five enriched items and their enriched genes of BP, CC, MF, and KEGG, respectively. BP, biological process; CC, cellular component; MF, molecular function; GO, Gene Ontology; KEGG, Kyoto Encyclopedia of Genes and Genomes; DESRGs, differentially expressed senescence-related genes.

Utilizing the clusterProfiler R package, we conducted GO functional annotation and KEGG pathway enrichment analyses to better understand the functions of DESRGs. Three annotation databases of GO terms were used: Biological Process (BP), Molecular Function (MF) and Cellular Component (CC). Fig 5 showed the top 5 enriched GO terms and KEGG items. For BP, DESRGs exhibited significant enrichment in processes such as mitotic cell cycle phase transition, regulation of cell cycle phase transition and regulation of mitotic cell cycle phase transition. The MF of DESRGs were mainly enriched in protein serine/threonine kinase activity and protein serine kinase activity. For CC, the DESRGs were mainly enriched in chromosomal region, condensed chromosome and spindle pole. Additionally, the KEGG pathway enrichment analysis highlighted a significant association with Cellular Senescence. Fig 6 illustrates the enriched genes that correspond to BP, CC, MF, and KEGG.

### Constructing the PPI network and identifying hub DESRGs

**Fig 7:**
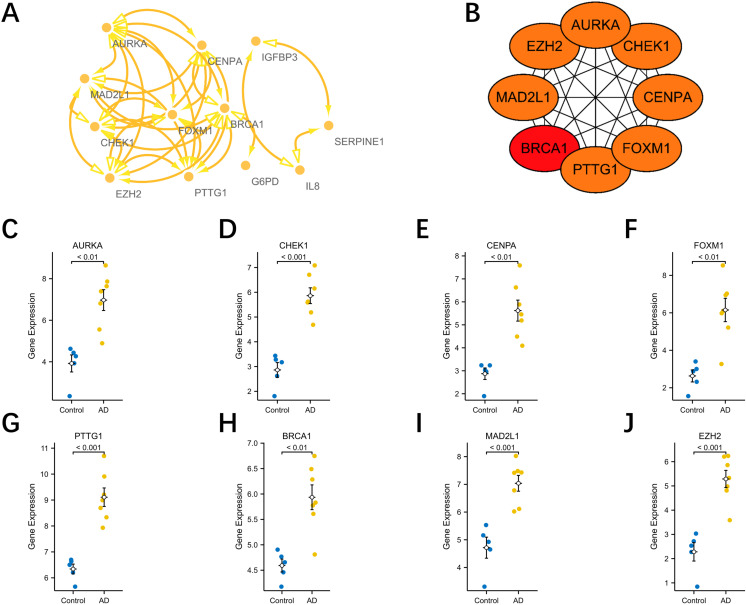
Protein-protein interaction network and hub genes. (A) Protein-protein interaction network constituted with the DESRGs; (B) Top 10 hub genes. Darker colors indicate a higher value. DESRGs, differentially expressed senescence-related genes.

The STRING online database was utilized to build a PPI network of integrated DESRGs, considering only interacting proteins. The PPI network comprised 12 nodes and 32 edges finally (Fig 7A). Subsequently, the CytoHubba plugin within the Cytoscape software facilitated the identification of the top 8 hub DESRGs, determined by their connectivity degree (Fig 7B). The expression levels of the top-eight hub DSERGs within the GSE52093 dataset were showed in Fig 7C–J.

### Validation of diagnostic values of DESRGs

**Fig 8:**
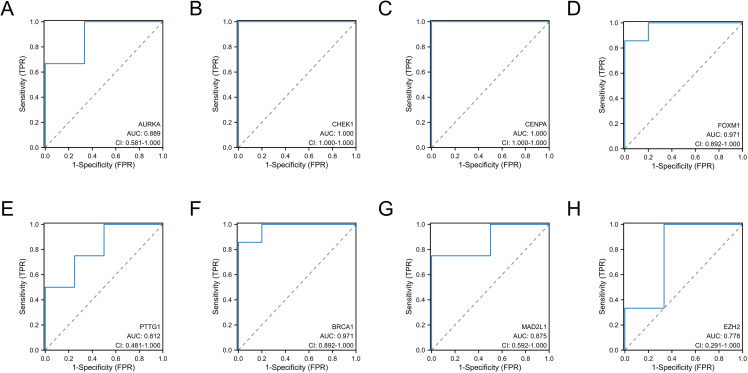
Diagnostic value of the 8 hub genes in the GSE52093 dataset. (A) ROC curves of AURKA, (B) CHEK1, (C) CENPA, (D) FOXM1, (E) PTTG1, (F) BRCA1, (G) MAD2L1, (H) EZH2. ROC, receiver operating characteristic; TPR, true positive rate; FPR, false positive rate.

**Fig 9:**
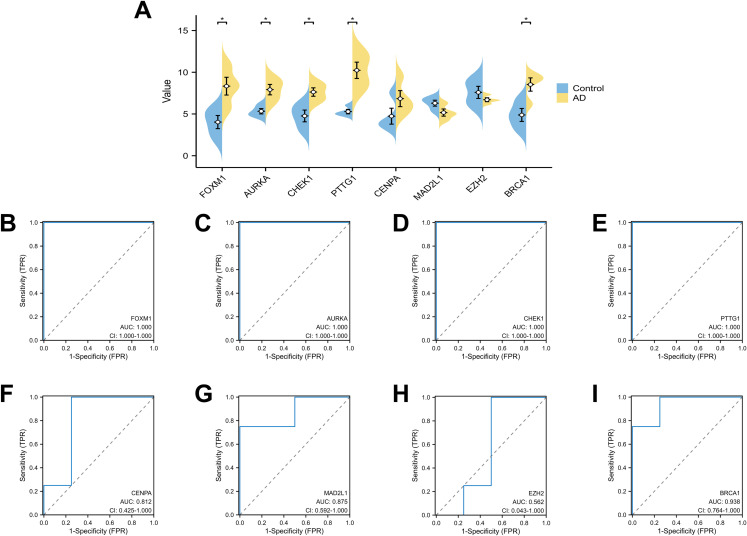
Expression and diagnostic value of the 8 hub genes in the GSE190635 validation dataset. (A) Expression levels of 8 hub genes in the AD and control samples were visualized by pods plot. (B) ROC curves of FOXM1, (C) AURKA, (D) CHEK1, (E) PTTG1, (F) CENPA, (G) MAD2L1, (H) EZH2, (I) BRCA1. ROC, receiver operating characteristic; TPR, true positive rate; FPR, false positive rate; *p < 0.05.

To validate the diagnostic value of the top eight hub DESRGs acquired from the earlier analysis, ROC curves were plotted, and the AUC value was employed to evaluate their efficiency in distinguishing AAD from control samples. As shown in Fig 8, the best diagnostic DESRGs were CHEK1 (AUC:1.000) and CENPA (AUC: 1.000), followed by FOXM1 (AUC:0.971), BRCA1 (AUC:0.971), AURKA (AUC:0.889), MAD2L1 (AUC:0.875), PTTG1 (AUC:0.812) and EZH2 (AUC:0.778). Then, the expressions of the hub DSERGs were extracted from the validation dataset, as illustrated in Fig 9A. Additionally, the diagnostic ability of hub DESRGs was also validated in the same dataset (Fig 9B–I) and the result indicated that FOXM1, AURKA, CHEK1 and PTTG1 had the highest diagnostic ability (AUC:1.000) followed by CENPA (AUC:0.812), MAD2L1 (AUC:0.875), BRCA1(AUC:0.938) and EZH2 (AUC:0.562).

### Construction of the miRNA-gene regulatory network

**Fig 10:**
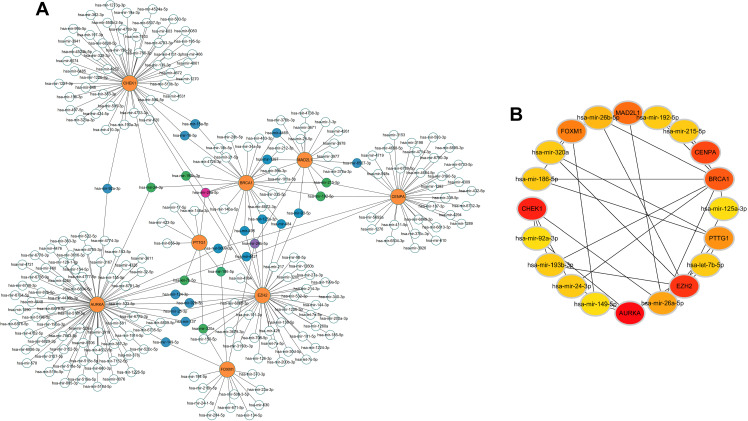
Integrated miRNA-DESRGs network for the top 8 hub genes. (A) Coexpression network constituted with the hub genes and target miRNAs. Orange circles represent hub genes. White circles represent miRNA which has the lowest connectivity with hub genes; Blue circles represent miRNA which has low connectivity with hub genes; Green circles represent miRNA which has moderate connectivity with hub genes; Purple circles represent miRNA which has high connectivity with hub genes and pink circles represent miRNA which has the highest connectivity with hub genes. (B) Hub miRNAs. Darker colors indicate a higher value. miRNA-DESRGs, microRNA- differentially expressed senescence-related genes.

NetworkAnalyst 3.0 (http://www.networkanalyst.ca/), a visual online platform aiding in identifying miRNA-gene interactions within Gene Regulatory Networks, was utilized to predict the target miRNAs for the eight hub DSERGs. miRNAs with a degree cutoff value = 1.0 were found for each hub DSERG. Finally, the eight hub DSERGs and targeted miRNAs were visualized via Cytoscape (version 3.9.1). Ultimately, we obtained 269 miRNAs of the 8 DSERGs (S3 Table). Among them, CENPA was regulated by 35 miRNAs, MAD2L1 was regulated by 17 miRNAs, FOXM1 was regulated by 14 miRNAs, CHEK1 was regulated by 52 miRNAs, BRCA1 was regulated by 26 miRNAs, PTTG1 was regulated by 8 miRNAs, AURKA was regulated by 73 miRNAs and EZH2 was regulated by 44 miRNAs. Fig 10A showed the miRNA-gene network, comprising 277 nodes and 269 edges and Fig 10B showed the hub miRNAs which were screened by the CytoHubba plugin in Cytoscape.

### Analysis of immune cell infiltration

**Fig 11:**
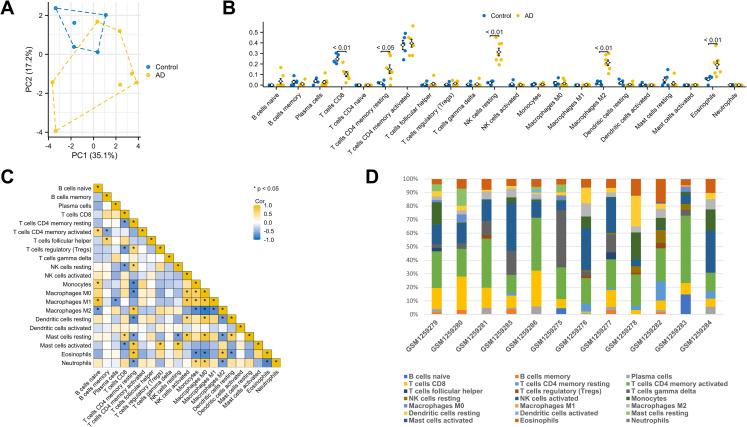
Visualization of immune cell infiltration between AD and control group. (A) PCA plot of immune cell infiltration between CL infection and control group samples; (B) The difference of immune infiltration proportions between AD and control samples. (C) Correlation heat map between different kinds of immune cells. The abscissa and ordinate represent different immune cells; different colors represent different correlation coefficients (yellow: positive correlation; blue: negative correlation). The darker the color, the stronger the relation. (D) Histograms of the contents of 22 types of immune cells in each sample. AD, aortic dissection.

**Fig 12:**
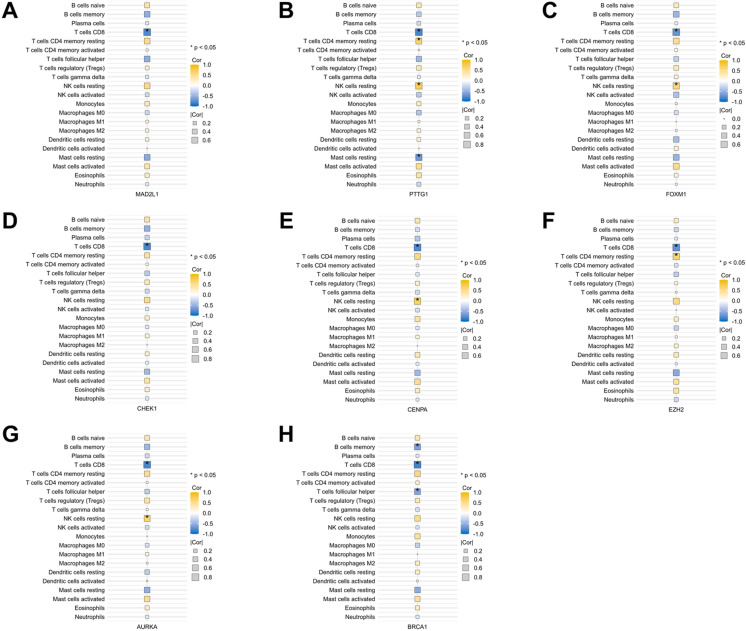
Analysis of correlation between hub genes and immune cells. (A) MAD2L1, (B) PTTG1, (C) FOXM1, (D) CHEK1, (E) CENPA, (F) EZH2, (G) AURKA and (H) BRCA1.

CIBERSORT website was utilized for analyzing immune cell infiltration. First, PCA indicated a noteworthy distinction in immune cell infiltration between the AD and control groups (Fig 11A). The scatter plot illustrated the presence of 5 significantly distinct immune cell types within the GSE52093 expression matrix following the Wilcoxon rank-sum test (Fig 11B). The AD group exhibited a higher proportion of CD4 memory resting T cells (p < 0.05), resting NK cells (p < 0.01), M2 macrophages (p < 0.01), and Eosinophils (p < 0.01) compared to the control group. Conversely, the control group manifested a greater fraction of CD8 T cells (p < 0.01). Furthermore, correlation analysis among 22 types of immune cells was performed. The results in Fig 11C indicated that CD4 memory resting T cells and Eosinophils showed the most synergistic effect (R = 0.85, P < 0.05). Meanwhile, M2 macrophages and M0 macrophages showed the most competitive effect (R = −0.87, P < 0.05). Fig 11D comprehensively showed the composition of immune cells in different samples. Last, to delve deeper into the immune mechanism involved in AAD development, we performed Spearman’s rank correlation analysis between the hub genes and immune cells. As shown in Fig 12, MAD2L1 displayed a negative correlation with CD8 T cells. PTTG1 exhibited a positive correlation with CD4 memory resting T cells, resting NK cells, while displaying a negative correlation with CD8 T cells and resting Mast cells. FOXM1 and AURKA both demonstrated a negative correlation with CD8 T cells and a positive correlation with resting NK cells. CHEK1 was negatively correlated with CD8 T cells. CENPA was negatively correlated with CD8 T cells and positively correlated with resting NK cells. EZH2 was negatively correlated with CD8 T cells and positively correlated with CD4 memory resting T cells. BRCA1 was negatively correlated with memory B cells, CD8 T cells and follicular helper T cells.

## Discussion

AD stands as among the most damaging cardiovascular diseases, frequently culminating in fatalities when diagnosis and treatment are not promptly administered. It involves the separation of the aortic wall due to intimal injuries, causing blood to flow into the aortic wall through the tear in the intima. Acute Type A Aortic Dissection represents the severest form, categorized as Stanford-A when involving the ascending aortic thoracic tract or the arch [12]. Data from the International Registry of Acute Aortic Dissection reveal a staggering mortality rate of approximately 2% per hour within 48 hours of onset in untreated AAD patients, with a 90% mortality rate within three months. Treatment with pharmacotherapy like β-blockers results in nearly 20% mortality within 24 hours and approximately 30% within 48 hours of AAD onset [1]. Rapid surgical intervention reduces the mortality rate to 10% within an hour and approximately 20% within a month [13]. These statistics highlight the limited efficacy of current pharmacological therapies for AAD, while surgical treatment is a demanding technique with high technical requirements and a long operative time and may lead to high rates of complications [14]. It is therefore crucial to identify new and more effective therapeutic strategies to defeat AAD.

Epidemiological studies have confirmed that aging is an independent risk factor for atherosclerotic cardiovascular disease (ASCVD) and related phenotypes [15]. Cellular senescence is an irreversible growth arrest induced by diverse intrinsic and extrinsic stresses, serving as a hallmark in both aging and aging-related diseases [16,17]. Recent years, considerable attention has been dedicated to identifying effective biomarkers that signify cellular senescence, especially in the cancer related studies [18]. However, the quest for diagnostic biomarkers of cellular senescence in AAD remains largely uncharted. In addition, previous studies on AAD have solely identified hub genes without comprehensive exploration of the fundamental molecular mechanisms driving AAD occurrence and development, or the specific roles of these hub genes in associated mechanisms [9,10]. Therefore, our study delves not only into screening cellular senescence-related diagnostic biomarkers of AAD patients but also innovatively explores immune infiltration in AAD. Moreover, we analyzed the correlation between immune cells and hub DESRGs to gain deeper insights into the pathological mechanisms.

In the present study, 20 DESRGs were identified using 700 DEGs and 279 SRGs, including 11 genes that induced senescence effects and 9 genes that inhibited senescence effects (Table 1). The subsequent KEGG, GO functional enrichment analyses indicated that the 20 DESRGs were enriched in the process of Cellular senescence, mitotic cell cycle phase transition protein serine, threonine kinase activity chromosomal region and chromosomal region. Through the CytoHubba plugin in Cytoscape software, we identified the top 8 hub genes in PPI networks, namely CHEK1, CENPA, FOXM1, BRCA1, AURKA, MAD2L1, PTTG1, EZH2.Three of these genes—CHEK1, FOXM1, and BRCA1—demonstrated particularly high diagnostic potential, with AUC values exceeding 0.9 in both training and validation datasets. The correlation between these genes and immune cell infiltration was also explored, revealing that CD8 T cells were significantly decreased, while NK cells and macrophages were elevated in AAD tissues compared with normal tissues.

Checkpoint Kinase 1 (CHEK1) is a serine/threonine-specific protein kinase belonging to the CHEK family, involved in inducing cell cycle arrest upon DNA damage [19]. Studies in oncology revealed that CHEK1 exhibits alterations in 0.80% of all cancers and mutations in 2.62% of malignant solid tumors [20]. What is more, CHEK1 expression has implications in several non-neoplastic diseases, such as Alzheimer’s disease [21], multiple myeloma [22], autoimmune disease [23,24] and so on. A recent study from Nature [25] showed that the abnormal expression of the CHEK1 will promote the accelerated aging of women’s ovaries and previous studies have shown that the loss of ovarian health will promote women’s systemic aging, which will lead to increased risk of cardiovascular disease, bone disease and neurodegenerative diseases and eventually shorten women’s life expectancy [26]. Its role in cancer and other diseases has been widely studied, however, this is the first study to propose CHEK1 as a potential biomarker for AAD. Hence, by investigating the correlation between aging and AAD, we noted a substantial increase in the expression of the SRG CHEK1 among AAD patients, and further studies highlighted CHEK1’s potential utility as a diagnostic marker in AAD.

FOXM1, part of the Forkhead domain protein family, exhibits expression exclusively in proliferating cells and has critical functions in cell-cycle progression [27]. Multiple studies have revealed FOXM1’s significant role in regulating oxidative stress, contributing to malignant transformation and tumor cell survival, considering its overexpression in various human malignancies [28]. Vast majority of research on FOXM1 consistently highlights its high expression correlating with radio and chemoresistance, which may lead to unfavorable patient outcomes in various cancers such as breast cancer [29], small cell lung cancer [30]. FOXM1 also exerts a pivotal role in the vascular system. Various factors originating from dysfunctional endothelial cells induce FOXM1 expression in smooth muscle cells (SMCs) and trigger FOXM1-dependent SMC proliferation, which contributes to atherosclerosis, aortic aneurysm, pulmonary vascular remodeling and pulmonary hypertension [31,32]. In additional to this, FOXM1 inhibitor showed high efficacy in blocking neointima formation in animals model [33], suggesting that targeting the FOXM1 axis could be a viable therapeutic approach in preventing restenosis [34]. In this study, we observed a significant increase in the expression of the SRG FOXM1 in AAD patients, and combining the results above, we proposed that FOXM1 might play a proatherogenic role in AD development and could potentially serve as a diagnostic marker. However, confirming this hypothesis requires further extensive research and investigation.

BRCA1, breast cancer gene 1, known as a tumor suppressor gene, is directly linked with hereditary breast cancer [35]. It exerts multiple effects on DNA repair and affords resistance against cellular stress responses [36]. Available evidence obtained from studies on animal models and human BRCA1 mutation carriers showed a correlation of BRCA1 deficiency with various cardiovascular diseases, such as ischemic heart disease, atherosclerosis, and cardiac muscle disorders linked to chemotherapy [37,38]. In human atherosclerotic carotid artery plaque samples, BRCA1 levels were found to be decreased compared to adjacent normal tissues [39]. All results presented above showed that BRCA1 may play a protective role in cardiovascular disease and may be a represent potential therapeutic target to improve endothelial dysfunction and retard atherosclerosis. However, our current findings showed a significant upregulation of the SRG BRCA1 in AAD patients. Unlike the previous studies, it seemed to play a disease-driving role in the development and progression of AAD. Despite this, these studies collectively highlight the significance of BRCA1 in cardiovascular disease or atherosclerosis and moreover, our findings proposed BRCA1 may as a potential biomarker for diagnosing AAD.

Multiple studies have highlighted the association between abnormal miRNA expression and the pathological mechanisms of AD [40,41]. MiRNAs play a significant role in regulating the proliferation, apoptosis, senescence, and migration of vascular smooth muscle cells (VSMCs) in AD [42]. For example, overexpressing miR-107-5p in RASMC cells promoted the cell proliferation and inhibited the cell apoptosis, indicating an inhibitory effect on the progression of AD [43]. miRNA-22 inhibits VSMCs apoptosis during AD vascular remodeling by targeting p38 mitogen-activated protein kinase α(p38MAPKα) [44] and miR-146a-5p enhances VSMCs proliferation and migration by targeting SMAD4 [45]. In the present study, 12 hub miRNAs were identified from the coexpression network of hub DESRGs and target miRNAs. Among them, miR-26a-5p was a common target miRNA of 5 hub DESRGs (CHEK1, PTTG1, BRCA1, MAD2L1, EZH2). miR-26a-5p, a member of the microRNA-26 family, is a conserved small RNAs with identical sequences at the seed region [46]. Previous studies showed that miR-26 members play key roles in cardiovascular diseases, neurological diseases, and malignant tumors [47,48]. Moreover, in vivo studies also showed that overexpression of miR-26a-5p potently inhibited VSMCs proliferation and migration, and dramatically prevented hypoxia-induced vascular remodeling [49]. Thus, combining with previous studies, we hypothesized that miR-26a-5p might play a crucial role in the pathological process of AD, potentially serving as an appealing biomarker for the diagnosis and treatment of AD. However, more research is necessary to support this hypothesis.

Considering few studies have reported immune filtration profile of AAD, and in conjunction with the GO, KEGG, and GSEA enrichment results, we observed significant involvement of DEGs in diverse immune responses. Therefore, we conducted the analysis of immune infiltration of AAD and explored the correlation between hub DESRGs and the infiltration of immune cells. Though immunohistochemistry is the standard method for assessing immune cell infiltration in tissues [50], quantifying and comparing different cell subpopulations still present significant challenges. While flow cytometry is another method for quantitative assessment, it has limitations with limited sample amounts [51]. In contrast, CIBERSORTx analysis was reported as an effective tool for examining immune cell landscapes and offer new insights into the neoplastic disease pathogenesis [52].

In the present study, CIBERSORT evaluation showed a distinct variance in immune cell infiltration between the AD and normal groups. Assessing immune cell subsets and determining the overall immune infiltrate in each sample through CIBERSORT typically reflect the time course of innate and adaptive immune responses in human disease [53]. Notably, compared with that in normal tissues, the fraction of CD8 T cells was significantly decreased in AAD tissues, while NK cells and macrophages was significantly increased in AAD tissues and the proportions of the NK cells and macrophages were significantly higher in each sample of AAD. Moreover, correlation analysis also showed a positive correlation between the 8 hub DESRGs and CD8 T cells as well as NK cells. A mountain of evidence have indicated the crucial role of macrophages in aortic wall inflammation and their involvement in AD [54]. Cytokines derived from macrophages upregulate Fas-associated proteins in VSMCs through intricate cellular interactions, inducing apoptosis and reducing VSMCs, consequently compromising the arterial wall integrity [55]. Macrophages are the primary immune cells infiltrating the aortic media and adventitia, initiating a localized inflammatory response after dissection, and several complications of AAD are also associated with them [54,56]. Indeed, macrophages have been demonstrated to cause apoptosis in SMCs, degrade elastic fibers, and induce neovascularization, contributing to the deterioration and separation of the aortic wall [57]. Briefly, our findings, combined with the published scientific literature showed that the high expression of macrophages contributed to aortic dissection through involvement in the immune-inflammatory response, implying macrophage could be emerging as a new target for AAD.

CD8 T cells play crucial roles in innate and adaptive immunity, defending against both external threats, such as viral infections, and internal abnormalities like tumor cells or aberrant cells infected by pathogens [58]. CD8 T cell expression exerts protective effects against AD by diminishing immune cell activation, elevating interleukin IL-10 expression, and reinforcing its anti-inflammatory effects, thus, in turn, mitigating the inflammatory response in vessels and preventing the AD development [59,60], which is consistent with our findings. Additionally, using flow cytometry experiments, Flavia et al. observed an elevation in NK cells and macrophages, concomitant with a decline in T helper fractions among patients with AAD [61]. Feng et al. delineated the immune cell landscape of TAAD using the ssGSEA algorithm, highlighting the dominance of innate immune-associated cells—specifically, macrophages, activated dendritic cells, and NK cells—over adaptive immune-related cells in AAD tissues [62]. All these results suggested that the elevated presence of NK cells and macrophages, coupled with decreased CD8 T cells appear to prevent development and progression of AD.

Our study not only innovatively first reported the linkage between eight senescence-related genes and AAD progression, but also employed the CIBERSORT algorithm to analyze immune infiltration in AD and explored the correlation between hub genes and infiltrating immune cells. Our findings revealed a significant correlation between key senescence-related genes (CHEK1, FOXM1, BRCA1) and immune cell infiltration in AAD. Specifically, reduced CD8 T cell levels and increased NK cell and macrophage populations in AAD tissues align with previous reports highlighting the predominant role of innate immune responses in AAD pathogenesis. Macrophages contribute to vascular inflammation by promoting smooth muscle cell apoptosis and extracellular matrix degradation, thereby exacerbating aortic wall fragility. CHEK1 and FOXM1 may influence this process by modulating immune cell recruitment and activation, as suggested by their strong associations with macrophage markers. Moreover, a comparison with prior studies [9,33,54] indicates that our integrated approach—combining gene expression profiling, immune infiltration analysis, and diagnostic validation—offers a more comprehensive understanding of the molecular and immune mechanisms underlying AAD. By identifying potential biomarkers with high diagnostic accuracy (AUC > 0.9), such as CHEK1 and FOXM1, our study provides a foundation for future translational research and therapeutic development. However, several limitations of our study should be noted. Primarily, our research focused solely on cellular senescence-related genes from the CellAge database, potentially overlooking additional yet undiscovered cellular SRGs. Second, despite conducting external validations to address this limitation, the sample sizes in each dataset were relatively small. Third, while the three diagnostic genes we screened are linked to cellular senescence, there is inadequate research confirming their precise role in regulating senescence in AAD. Finally, this study solely relies on bioinformatic analysis utilizing existing databases and lacks experimental validation. Hence, animal models should be developed to validate the potential impact of immune dysregulation on disease progression. What’s more, cell experiments are warranted to dissect the molecular mechanisms of the eight genes involved in AAD.

## Conclusion

Briefly, we identified and validated eight SRGs as new diagnostic biomarkers during the early stages of AAD, among which CHEK1, FOXM1, BRCA1 exhibit particularly high diagnostic value. These genes potentially exert a substantial influence on the occurrence and progression of AAD through their impact on inflammatory responses or immune regulation. As a result, our findings suggest promising avenues for AAD treatment.

## Supporting information

S1 Table Comprehensive list of all SRGs. (XLSX)

S2 Table A total of 700 DEGs. (XLSX)

S3 Table 269 miRNAs of the 8 DSERGs. (XLSX)
